# Surfactant-Mediated Airway and Acinar Interactions in a Multi-Scale Model of a Healthy Lung

**DOI:** 10.3389/fphys.2020.00941

**Published:** 2020-08-14

**Authors:** Haoran Ma, Hideki Fujioka, David Halpern, Donald P. Gaver

**Affiliations:** ^1^Department of Biomedical Engineering, Tulane University, New Orleans, LA, United States; ^2^Center for Computational Science, Tulane University, New Orleans, LA, United States; ^3^Department of Mathematics, University of Alabama, Tuscaloosa, AL, United States

**Keywords:** biofluid mechanics, surfactant, multi-scale modeling, fluid–structure interactions, high-performance computing, acute respiratory distress syndrome

## Abstract

We present a computational multi-scale model of an adult human lung that combines dynamic surfactant physicochemical interactions and parenchymal tethering between ~16 generations of airways and subtended acini. This model simulates the healthy lung by modeling nonlinear stress distributions from airway/alveolar interdependency. In concert with multi-component surfactant transport processes, this serves to stabilize highly compliant interacting structures. This computational model, with ~10 k degrees of freedom, demonstrates physiological processes in the normal lung such as multi-layer surfactant transport and pressure–volume hysteresis behavior. Furthermore, this model predicts non-equilibrium stress distributions due to compliance mismatches between airway and alveolar structures. This computational model provides a baseline for the exploration of multi-scale interactions of pathological conditions that can further our understanding of disease processes and guide the development of protective ventilation strategies for the treatment of acute respiratory distress syndrome (ARDS).

## Introduction

The lung is an extraordinary example of a physiological organ whose stability and function depends critically upon multiscale geometric interactions and processes that interlink tissue and biofluid mechanics. Airflow during inspiration and expiration changes the lung volume, causing the geometric change of highly compliant pulmonary tissues. During this volume change, tissue-level interactions like parenchymal tethering and molecular level interactions like surfactant re-distribution take place simultaneously in order to stabilize the lung. The lung's large surface area to volume ratio suggests the great importance of surfactant-mediated surface tension to lung stability. Meanwhile, the mechanical interdependency of lung units provides parenchymal tethering support for compliant airways that is instrumental to stabilizing the lung by maintaining adequate airflow to foam-like acinar structures that, in turn, support compliant airways. The large liquid-covered surface of the pulmonary tissue requires surfactant transport to dynamically modulate the surface tension during volume cycling in order to prevent fluid–structure instabilities, as it reduces the work of breathing (West, 1977, 2012; Notter, 2000). In this paper, we describe the development of a multi-scale model of a healthy lung from which future studies of pathophysiological systems can be explored.

The computational investigations of lung mechanics have been conducted on different scales. For example, at the organ level, our model is based upon an algorithm developed by Tawhai et al. who presented a computational method for generating the conducting airway network (Tawhai et al., 2000). At the tissue level, Lambert et al. (1982) and Fujioka et al. (2013) have developed computational models to describe the non-linear properties of the acinus and conductive airways, respectively. Micro-mechanical models of parenchyma support are based upon the recent work by Ryans et al. (2019), that is based on the foundational work of Wilson (1972), Lai-Fook et al. (1977, 1978), and Anafi and Wilson (2001). Surfactant transport properties are based on multilayer transport processes described by Krueger and Gaver (2000), which models surfactant behavior originally described by Clements (1957), Horn and Davis (1975), Smith and Stamenovic (1986), and Schurch (1995).

While these and many other studies have provided useful information and analysis about pulmonary mechanics on separate scales, the interactions between all of these interlinking processes have not been modeled directly. Ryans et al. demonstrated a reduced-dimension modeling approach for investigating the recruitment/de-recruitment of the airways without full fluid–structure interactions or surfactant transport processes (Ryans et al., 2016). Wall et al. (2010) developed a 3-D lung model with CT-based geometries up to a maximum of approximately seven generations, including not only airway wall deformability but also the influence of surrounding lung tissue. Filoche et al. (2015) presented a mathematical model of surfactant replacement therapy in a 3-D lung structure to investigate whether the instilled surfactant mixture actually reaches the adult alveoli/acinus in therapeutic amounts. Interlinking the multiscale processes is likely to introduce forcing and response characteristics that are not investigated in isolated models of isolated scales. For example, the multilayer surfactant behavior has heretofore been modeled in an oscillating bubble system with a pre-defined bubble radius. In contrast, in the pulmonary system the radius of airways and acini are driven by an oscillating pleural pressure on the surface of the lung. This, in turn, induces a heterogeneous distribution of mechanical stresses within the lung that dynamically modifies the lung sub-structure. In this process, the geometry of the airways and alveoli are modified, which induces surfactant transport that modulates the surface tension of the lining fluid on the internal structures of the airways and alveoli and, once again, modulates the tethering pressure and geometry of the system (Perun and Gaver, 1995). These feedback processes, when fully modeled, may elucidate pulmonary behavior that cannot understood from the reductionist approach and may exhibit emergent behavior (Suki and Bates, 1985).

In this study, our goal is to build a computational model of an adult human lung that combines dynamic multi-scale interactions between ~16 generations of airways and subtended acini generated by a space filling algorithm, including airflow, multilayer surfactant transport and airway/alveolar interdependency. We explore this system under an oscillating pleural pressure that is applied to simulate the breathing of a healthy lung. Our goal is to develop this model as a useful tool to understand the multi-scale lung mechanics and a critical baseline for the future study of the pathological conditions.

## Materials and Methods

### Conceptual Formulation

#### Framework

We have developed a rational engineering design approach for elucidating the surfactant physicochemical interactions and the mechanical interdependency within a healthy lung. It is assumed that under healthy conditions, the pleural pressure (*P*_*PL*_) is always negative and varies with time during the normal breathing cycle. The framework of this simulation approach is based upon three components:

- Tissue Components represent the series and parallel segments of airways and acini. The 3-D structure of airways and acini is determined using an anatomically based space-filling algorithm (Tawhai et al., 2000) in order to introduce inter-connections between airways and acini via parenchymal tethering, which is heterogeneously distributed.
- Air Components represent the volume of the air spaces, with temporal changes based upon the flow rate within each segment. Segmental flow rates are determined by regional pressure differences and the status of each of the compliant airways and acini as determined from the Tissue Component.
- Liquid Components represent the lining fluid and surfactant in the airways and acini, which dynamically modulate the surface tension. This, in turn, influences the Tissue Component stresses and thus affects flow rate.

#### Interactions Among Components

**Figure 1:**
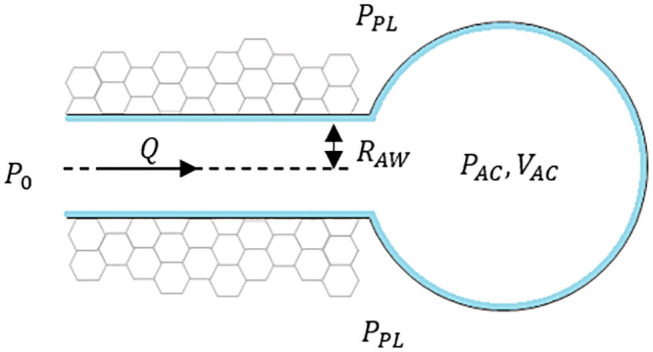
Schematic of a “simple” lung unit with one terminal airway attached to an acinus.

To illustrate the concept of interactions among the three components, consider the presence of a terminal airway surrounded by parenchyma and connected to an acinus at one end as seen in Figure 1, where *P*_*PL*_ is the pleural pressure, *P*_*AC*_ is internal acinar pressure, *R*_*AW*_ is the radius of airway, *Q* is the airflow toward the acinus. The pressure at the open end of the airway is considered a constant *P*_0_ to simplify this situation.

I. Tissue Components

These are defined by airways and acini, shown in simplified form in Figure 1. The components are defined by

- Airways: The dynamic radius of the airway is defined by the tube law (Lambert et al., 1982) as a function of the airway transmural pressure (*P*_*TM,AW*_), described conceptually as:
  (1) $$\begin{array}{l} P_{TM,AW}=\;P_{int,AW}\;-P_{ext,AW}, \end{array}$$
  where *P*_*int,AW*_ represents the internal pressure from the internal air pressure and the Laplace pressure drop from surface tension, and thus is modulated by Air and Liquid Components. *P*_*ext,AW*_ represents the external stresses from the time-dependent pleural pressure (*P*_*PL*_) and is modulated by the parenchymal tethering using a shear modulus (*G*_*eff*_) representation of the acinar support as described in Ryans et al. (2019).
- Acinus: Similarly, from Fujioka et al. (2013), the volume of acinus is a function of acinar transmural pressure
  (2) $$\begin{array}{l} P_{TM,AC}=\;P_{int,AC}-\;P_{ext,AC}, \end{array}$$
  where *P*_*int,AC*_ represents the internal pressure from the acinus air pressure and the Laplace pressure drop from surface tension within alveoli that create the acinus, and *P*_*ext,AC*_ = *P*_*PL*_.

In addition to structural coupling that exists through *P*_*int,AW*_ and tethering forces between the acini and the exterior of airways, internal coupling exists through internal pressures because *P*_*int,AC*_ is a function of the acinus volume. This sets *P*_*AC*_ as the end-condition of the Air Component system, which is necessary for determining the flow rates in the system, as described below.

II. Air Components

These are driven by airflow to and from airways and the subtending downstream acinus in the following manner:

- Airflow (*Q*): Determined by the pressure difference at the two ends of the airway (*P*_*AW*_), and the conductance (*C*) of the airway, *Q* = *CP*_*AW*_.
- Air Conductance (*C*): We simplify conductance as a function of the pressure-dependent airway radius, viscosity and length, following a Poiseuille relationship.
- Conservation of volume dictates that the flow determines the change of volume of the downstream acinus (*V*_*AC*_).

As can be seen from the above descriptions, the Air Components create an internal coupling to the Tissue components through *P*_*int,AW*_ and *P*_*int,AC*_.

III. Liquid Components

These determine the distribution of fluid in the airways and alveoli. Two fundamental aspects are imposed:

- Conservation of mass: The volume of liquid is conserved within each airway and acinus. So, as an airway or acinus changes shape, the liquid film thickness changes accordingly.
- Surfactant physicochemical interactions: Conservation of mass of surfactant is enforced. As the airway and alveolar surface areas change, this triggers multilayer surfactant transport (Krueger and Gaver, 2000). Through a surface tension equation of state, a dynamic surface tension exists and leads to a realistic full-lung pressure–volume loop.

As can be seen from these interactions, the liquid components directly influence *P*_*int,AW*_ and *P*_*int,AC*_ in Tissue Components.

#### System Evolution

The key to the modeling approach is the evolution of the system as each component interacts with each other. To evolve the system, the time-dependent pleural pressure *P*_*PL*_ (*t*) is prescribed and the rest of the system is updated with an adaptive time increment *t* from the current state. The new state after the time increment is solved by the method described in the Modeling Implementation section and the breathing cycle is tracked.

### Modeling Implementation

#### Modeling Overview

**Figure 2:**
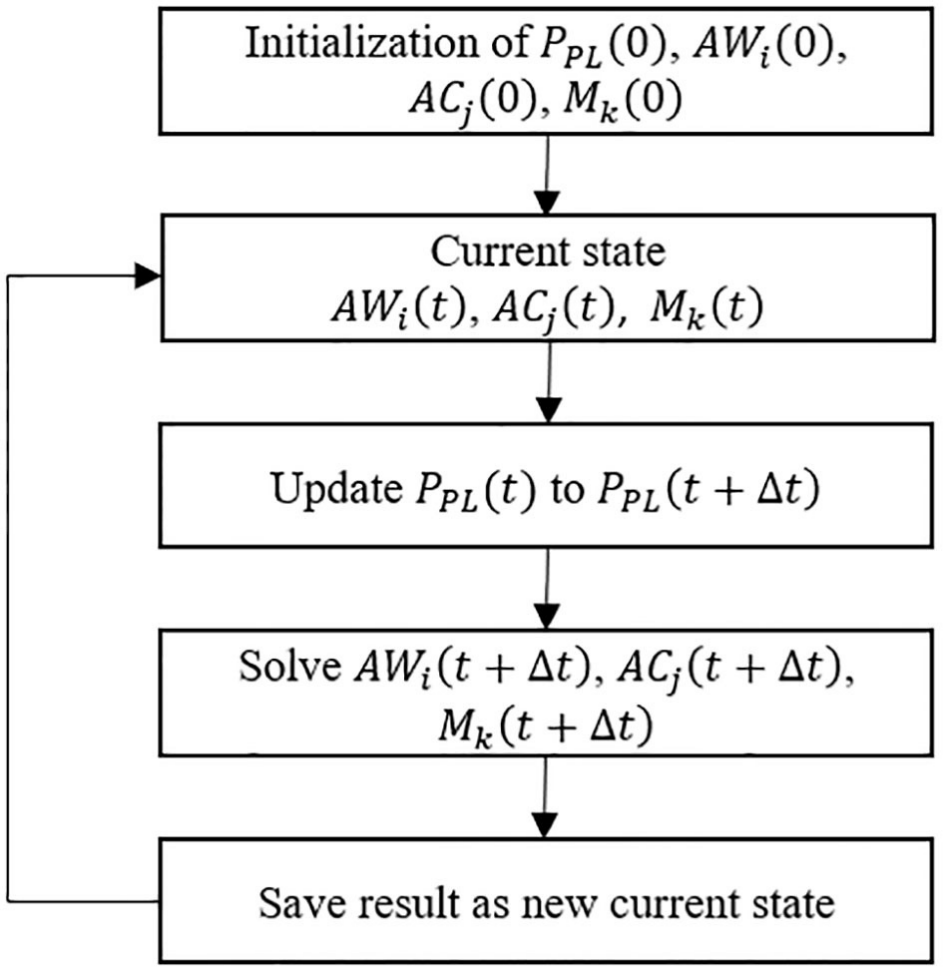
Schematic flow chart of the of system evolution for the simulation model.

To investigate surfactant-surface tension interactions and the mechanical interdependency within a healthy lung, the algorithm shown in Figure 2 was used to simulate the whole lung respiration. Initially the time-dependent pleural pressure *P*_*PL*_ (*t*) is prescribed at the beginning of each time step. This provides the driving pressure for ventilation, as exists for normal respiration. We define the fundamental solution vectors for the airways, acini and surfactant as *AW*_*i*_(*t*) = [*P*(*t*), *R*(*t*)]_*i*_, *AC*_*j*_(*t*) = [*P*(*t*), *V*(*t*)]_*j*_, and *M*_*k*_(*t*) = [*M*_*p*_(*t*), *M*_*s*_(*t*), *M*_*b*_(*t*)]_*k*_, respectively, where *P* is airway/acinus pressure, *R* is airway radius, *V* is volume of acinus, *M* is a vector consists of the surfactant masses in each region of the multilayer structure, including *M*_*p*_ for the primary layer, *M*_*s*_ for the secondary layer, *M*_*b*_ for the bulk liquid. Each airway or acinus is represented by two solution vectors, one of which is *AC*_*i*_(*t*) or *AW*_*j*_(*t*) containing tissue component properties, and the other is *M*_*k*_(*t*) containing liquid component properties. *AC*_*i*_(*t*), *AW*_*j*_(*t*) and *M*_*k*_(*t*) are solved simultaneously based on the mechanical relationship described in Network Flow Dynamics using a differential-algebraic equation systems solver.

#### Tissue Components

I. Airway Morphology

**Figure 3:**
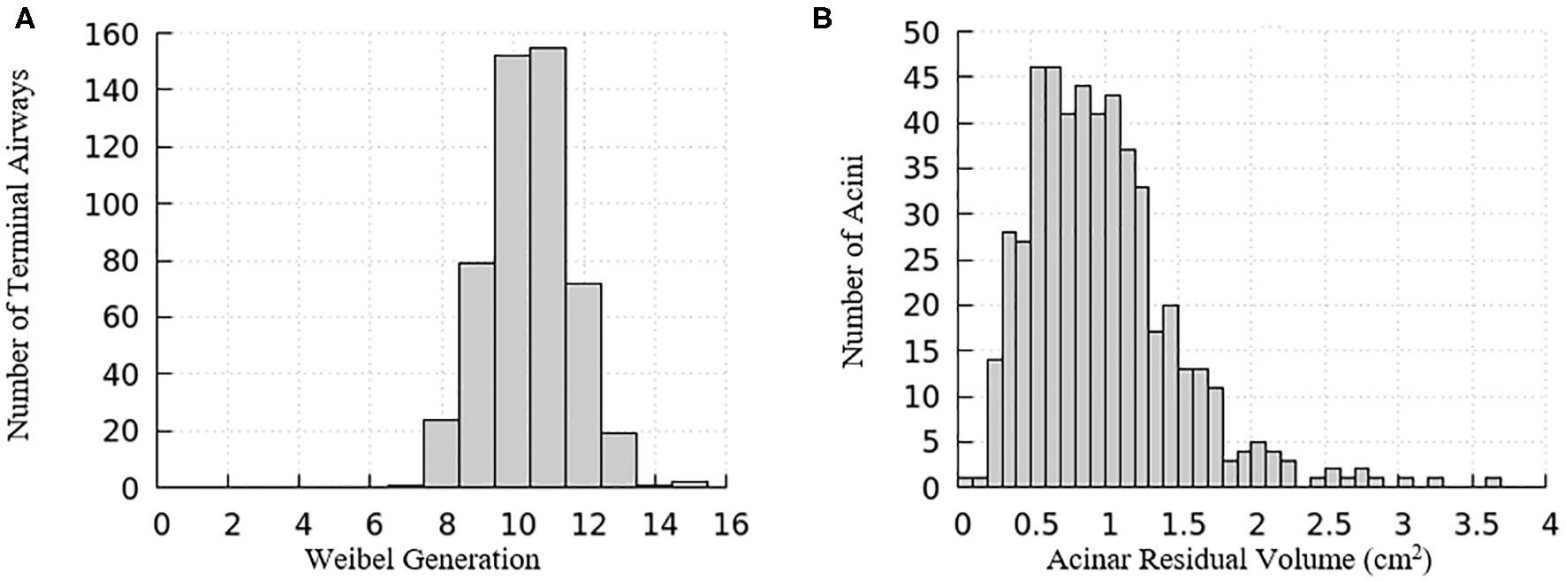
Distribution relationships for the computational model of a half-lung. **(A)** The Weibel generation of the terminal airways connecting to acinar regions, **(B)** the residual volume of the acini connecting to the terminal airways.

The domain of one half of the lung is generated using an anatomically based space-filling algorithm from Tawhai et al. (2000). It consists of a network of airways that has up to an equivalent Horsfield 15th generation (bottom-up with terminal airway defined as generation 1) that terminate into acinar regions with mechanical properties described in the “Acinus Component” section (Fujioka et al., 2013). The equivalent Weibel generation (top-down from trachea as generation 0) of terminal airways and the residual volume of their attached acini are shown in Figure 3. We note that structural differences exist among airways and acini, which will appear as important contributors to the heterogeneity in surface tension and parenchymal tethering described in the “Results and Discussion” section.

Airways and acini are positioned in a 3-D space in order to build inter-connections between the airways and acini (Tawhai et al., 2000). The morphology of the pulmonary airways was based on Lambert et al. by providing the airway maximum radius (*R*_*AW,max*_) and normalized cross-sectional area α at *P*_*TM,AW*_ = 0, i.e., $\alpha_{0}=\frac{A_{0}}{A_{max}}$ (Lambert et al., 1982).

II. Compliant Airway Components

**Figure 4:**
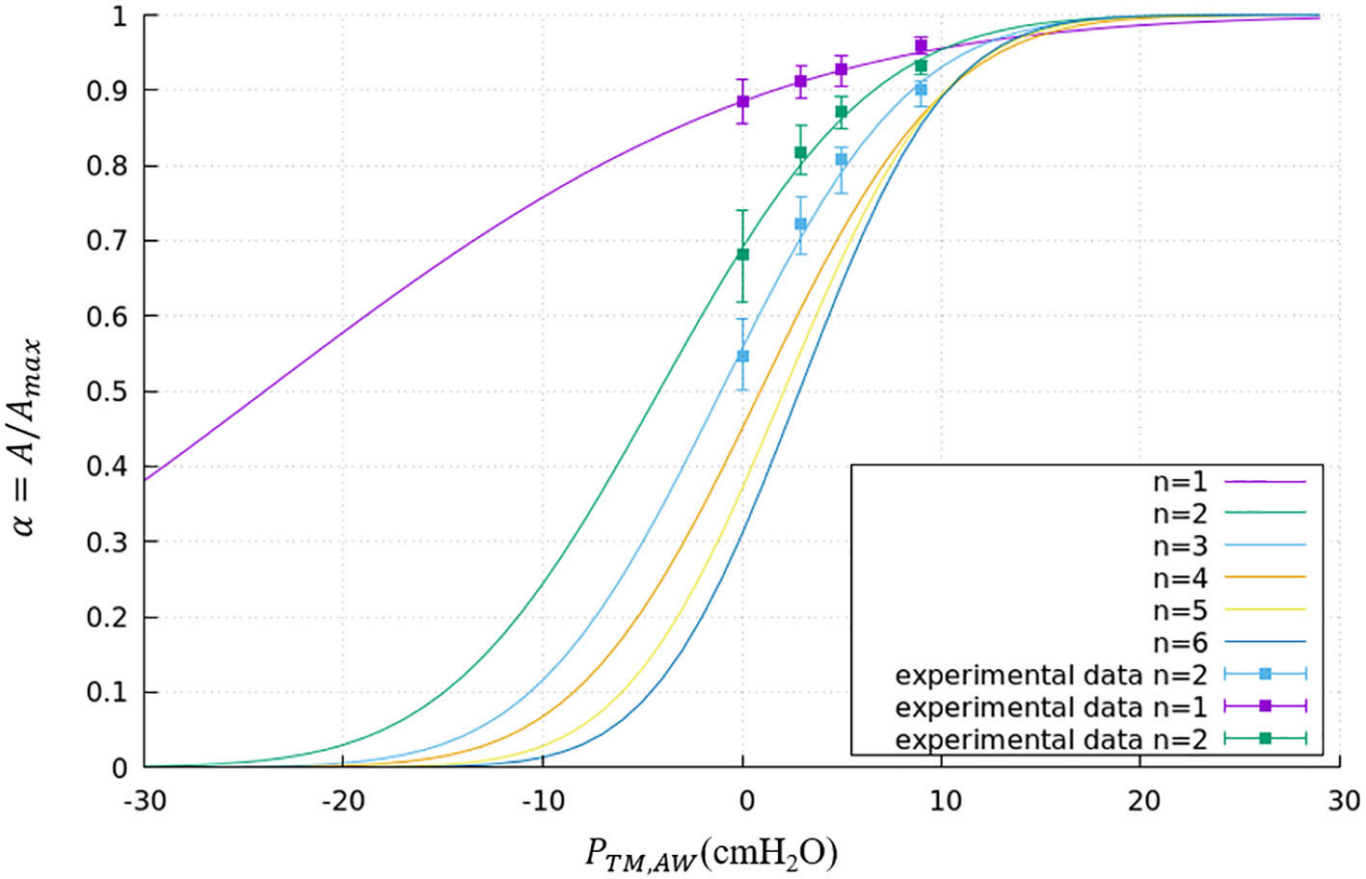
Tube law through the Weibel 6th generation. Airways smaller than the 6th generation are assumed to have the same tube law as the 6th generation. Experimental data is shown for the first 3 generations.

We used the airway morphological data to construct a tube law that describes the radius of a compliant airway as a function of the airway transmural pressure. For a healthy lung, we assume that the acinus is the most compliant region of the system, so that the parenchyma tethers the embedded airway open during inspiration, as described in the “Parenchymal Tethering” section, below. To do so, we defined a tube-law that follows a sigmoidal form that matches the airway-based morphological parameters. The general form of the tube-law is defined as

(3) $$\begin{array}{l} \begin{array}{c} \frac{R_{AW}}{R_{AW,max}}=\sqrt{0.5\times \left[1+\mathrm{erf}\left(aP_{TM,AW}+b\right)\right]}, \end{array} \end{array}$$

where *P*_*TM,AW*_ is the transmural pressure defined in Equation (1), *R*_*AW,max*_ is the maximum radius of this airway. The parameters a and b are generation-based constants shown in Table 1. The tube law behavior through the Weibel 6th generation as well as experimental data for the first 3 generations are shown in Figure 4 (Lambert et al., 1982).

**Table 1:** Generation-based constants used for the tube law used in Equation (3).

**Generation (*n*)**	***A***	***b***
1	0.0354	0.8486
2	0.0841	0.3504
3	0.0947	0.1004
4	0.0969	−0.0889
5	0.1115	−0.2347
6–15	0.1219	−0.3506

The internal airway pressure *P*_*int,AW*_ is subject to the airway pressure determined by the average of the upstream and downstream pressure as well as the Laplace pressure drop from the liquid lining of the airway. The Laplace pressure drop and airway pressure can be expressed respectively as

(4) $$\begin{array}{l} P_{int,AW}=P_{AW}-\frac{\gamma }{R_{in,AW}},\;and \end{array}$$

(5) $$\begin{array}{l} P_{AW}=\frac{P_{DN}+P_{UP}}{2}, \end{array}$$

where *P*_*AW*_ is the airway pressure, γ is the surface tension of the liquid lining, *R*_*in,AW*_ is the radius from the center line of the airway to the interior surface of the liquid lining, *P*_*DN*_ is the downstream pressure, *P*_*UP*_ is the upstream pressure. For the airway shape calculation, we assumed the pressure is uniform within an airway and determined the average pressure by Equation (5).

The external airway pressure *P*_*ext,AW*_ is subject to the pleural pressure and the parenchymal tethering from surrounding acini. We modeled tidal breathing by forcing the pleural pressure to be prescribed by a sinusoidally varying waveform with a 1:2 inspiration–expiration ratio that varies between −5 cmH_2_0 and −8.2 cmH_2_0 with a period of 5 s (Swan et al., 2012). The parenchymal tethering pressure is determined based on the work from Ryans et al. detailed in Parenchymal Tethering (Ryans et al., 2019). The peri airway pressure can be expressed as

(6) $$\begin{array}{l} P_{ext,AW}=P_{PL}+2G_{eff}\left(\frac{\Delta R}{R}\right), \end{array}$$

where *P*_*PL*_ is the pleural pressure, *G*_*eff*_ is the effective parenchyma shear modulus, $\frac{\Delta R}{R}$ is the fractional radius change from the “hole” radius of parenchyma as described below in Equation (12). From Equations (4) to (6), Equation (1) can be rewritten as

(7) $$\begin{array}{l} P_{TM,AW}\left(t\right)=\;P_{AW}-\;2G_{eff}\left(\frac{\Delta R}{R}\right)-P_{PL}\left(t\right)-\frac{\gamma }{R_{in,AW}\left(t\right)}, \end{array}$$

where the transmural pressure varies with time due to the change in pleural pressure and radius change of compliant airways.

To determine the length of airways, it is assumed that the length change of airways is proportional to the size change of their surrounding parenchyma. The parenchyma model is described in Parenchymal Tethering.

III. Acinus Component

The morphological information of the representative acinar regions connected to the terminal airways of the lung is based on the octahedral models depicted in Fujioka et al. (2013). We represent the behavior of a block of alveoli (~1,200 alveoli representing ~1.7 × 10^−3^ cc of tissue) by a pressure-volume relationship derived from simulation results of this block. We follow the form suggested by Venegas et al. (1985) that successfully models whole-lung mechanics. As such, the pressure-volume relationship of this model is governed by

(8) $$\frac{V_{AC}}{V_{RV}}=a+\frac{b}{1+\exp\left[-(P_{TM,AC}-c\right)/d]},$$

where *V*_*AC*_ is the acinar volume, *V*_*RV*_ is the acinar volume at residual volume (RV), *P*_*TM,AC*_ is the acinar transmural pressure defined in Equation (2), a–d are constants that describe the mechanical characteristics of the parenchyma. Since we add the dynamic surface tension to the Laplace pressure drop in our form of *P*_*TM,AC*_ in Equation (11), this structural component is based upon parameters describing the tissue behavior only (zero surface tension model) with a = 1.03, b = 4.95, c = 5.04, d = 1.02.

The external and internal acinar pressure are

(9) $$\begin{array}{l} P_{ext,AC}=P_{PL},\;and \end{array}$$

(10) $$\begin{array}{l} P_{int,AC}=P_{AC}-\frac{2\gamma }{R_{\bar{ALV}}}, \end{array}$$

where *P*_*AC*_ is the acinar pressure, *P*_*PL*_ is pleural pressure, γ is the surface tension of the liquid lining and $R_{\bar{ALV}}$ represents the mean radius of the interior surface of the alveolar lining fluid inside the acinus. At this stage, alveoli are assumed to be spherical. From Equations (9) and **(10)**, Equation (2) can be rewritten as

(11) $$\begin{array}{l} P_{TM,AC}\;=\;P_{AC}-\frac{2\gamma }{R_{\bar{ALV}}}-P_{PL}, \end{array}$$

IV. Parenchymal Tethering

We utilized the parenchyma model from Ryans et al. (2019) to describe the tethering pressure. In that model, a cylindrical “hole” is surrounded by an annular region of parenchyma. An airway is laminated inside this parenchymal hole. Without the laminated airway, the equilibrium hole radius is defined as *R*_*H*_ so that when the strain in the annular parenchyma is uniform, this yields *P*_*ext,AW*_ = *P*_*PL*_. For non-uniform strain, following Lai-Fook et al. (1978), we define the effective parenchyma shear modulus *G*_*eff*_ by

(12) $$\begin{array}{l} \frac{\Delta R}{R}=\frac{R_{AW}-R_{H}}{R_{H}}=\frac{P_{ext,AW}-P_{PL}}{2G_{eff}}, \end{array}$$

where *R*_*AW*_ is the airway radius, *P*_*ext,AW*_ is the peri-airway pressure, *P*_*PL*_ is the pleural pressure. From analyses performed by Ryans et al. (2019), we assume the equilibrium hole radius is proportional to the cubic root of the weighted average of acinus volume within a distance of 5*R*_*AW*,0_ from the airway axis, where *R*_*AW*,0_ is *R*_*AW*_ at zero transmural pressure. This behavior is consistent with the finite-element model by Ma et al. (2013). We also assumed the equilibrium hole radius *R*_*H,FRC*_ is equal to *R*_*AW,FRC*_ when the surrounding acinus volume is at the Functional Residual Capacity, *V*_*FRC*_. Since the exact value of FRC is unknown before the simulation, *R*_*H,FRC*_ and *R*_*AW,FRC*_ are estimated from the isolated airway and acini model. We note that the equilibrium hole radius setpoint at FRC is an assumption that should be experimentally validated and is related to positive strain-deviation of terminal airways, as described in section Parenchymal Tethering and Strain Deviation.

From computational simulations as described from Ryans et al., the effective parenchyma shear modulus *G*_*eff*_ is empirically determined as a function of mean transpulmonary pressure (Ryans et al., 2019). The transpulmonary pressure and effective parenchyma shear modulus can be expressed as

(13) $$\begin{array}{l} \bar{P_{TP}}=\bar{P_{ALV}}-P_{PL},\;and \end{array}$$

(14) $$\begin{array}{l} G_{eff}=Ae^{b\bar{{P_{TP}}^{3}}}, \end{array}$$

where $\bar{P_{TP}}$ is average transpulmonary pressure, $\bar{P_{ALV}}$ is the weighted average of surrounding acinar pressures within a distance of 5*R*_*AW*,0_ from the airway axis, *A* = 4.75 cmH_2_O, *B* = 5.17 × 10^−4^ cmH_2_O^−3^.

#### Air Component

I. Air Conductance

We assume that all air flows are laminar and fully developed. Airflow throughout the lung model is described using a Poiseuille relationship at each airway. From Poiseuille's law, the conductance of a compliant airway is prescribed as a function of its radius and length:

(15) $$\begin{array}{l} C_{AW}=\frac{\pi {R_{in,AW}}^{4}}{8\mu L}, \end{array}$$

where *C*_*AW*_ is the conductance of airway, *R*_*in,AW*_ is the radius from the center line of the airway to the interior surface of the liquid lining, μ is the viscosity of air, *L* is the length of the airway. We acknowledge that this is inaccurate when Reynolds numbers are high, as in the central airways (trachea and mainstem bronchi) and first generations of the model of the lung. We also neglect the loss of pressure at bifurcations.

II. Network Flow Dynamics

In our model, the axial component of the pressure gradient inside each airway is assumed to be a constant. We also assume that all air flows through airways are fully developed and without turbulence. The flow rates at the airway level are determined by

(16) $$\begin{array}{l} Q_{AW,i}=C_{AW,i}\left(P_{UP,i}-P_{DN,i}\right), \end{array}$$

where *Q*_*AW,i*_ is the flow rate of the airway, *C*_*AW,i*_ is the conductance of the airway, *P*_*UP,i*_ and *P*_*DN,i*_ are the upstream and downstream pressure of the airway, respectively.

At the terminal end of the system (acinus), the flow changes the acinus volume as

(17) $$\begin{array}{l} Q_{AW,i}=C_{AW,i}\left(P_{UP,i}-P_{AC,i}\right)=\frac{dV_{AC,i}}{dt}, \end{array}$$

where *P*_*AC,i*_ is the acinus pressure, *V*_*AC,i*_ is the acinus volume.

We assume that there is no pressure loss at the bifurcations. Conservation of flow rate at each bifurcation is satisfied by an equation of the form

(18) $$\begin{array}{l} Q_{AW,P}+Q_{AW,D1}+Q_{AW,D2}=0, \end{array}$$

where *P* stands for the parent airway, and *D* stands for the daughter airways.

Equations (16)–(18) are applied to each acinus, airway and bifurcation to build the differential-algebraic equation system. We note that we neglect the loss of pressure at the airway bifurcation (Filoche et al., 2015).

#### Liquid Components

I. Conservation of Liquid Phase

We assume the amount of liquid is conserved within each airway/alveolus. Thus the liquid film thickness has to varies with time due to changes in radius of airway/alveolus. A parameter which characterizes this change is defined as

(19) $$\begin{array}{l} \varepsilon =\frac{R_{out}-R_{in}}{R_{out}}, \end{array}$$

where *R*_*out*_ is the airway/alveolus radius, *R*_*in*_ is the radius from the center(line) to the liquid lining that determines the Laplace pressure drop. The change in the volume of the airways and alveolus therefore modulates the film thickness, which feeds back to the stress balance Equations (7) and (11). We note that the change of airway caliber as well as length is accounted for in the conservation of mass calculation for determining the dynamic film thickness.

II. Surfactant Physicochemical Interactions

**Figure 5:**
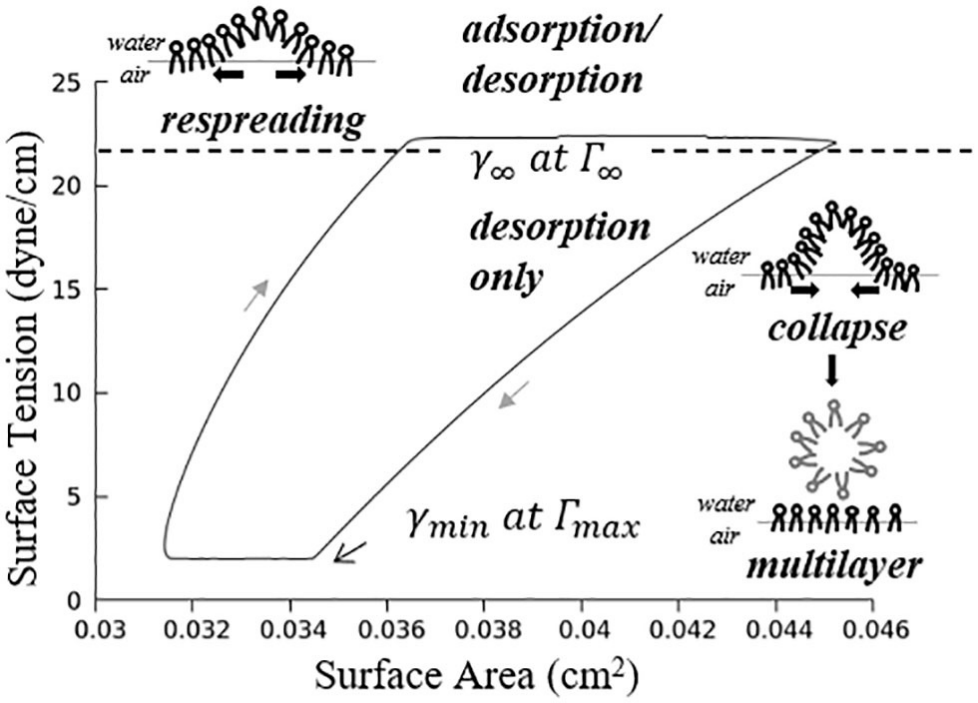
Example hysteresis loop of an alveolus. Γ_1_ is inversely proportional to the interfacial area. The primary layer begins to collapse when Γ_1_ ≥ Γ_∞_, forming a secondary layer between the bulk and primary layer. When Γ_1_ < Γ_∞_, the secondary layer dynamically respreads, which stabilizes the surface tension.

The mechanism of surfactant transport was based on the multilayer surfactant behavior analysis done by Krueger and Gaver (2000). Soluble surfactant in the liquid lining of airways/acini exists in the bulk fluid and two surface layers: primary and secondary layers. The primary layer is in direct contact with the air-liquid interface, while the secondary layer is created by collapse of the primary layer and resides between the primary layer and the bulk fluid. Adsorption/desorption can only happen between the bulk liquid and the primary layer. Only the surfactant in the primary layer can reduce the surface tension. The surface tension of water $\gamma_{0}=72\frac{dyne}{cm}$. From Krueger and Gaver (2000), the equilibrium surface tension $\gamma_{\infty }=22\frac{dyne}{cm}$ at the equilibrium surfactant concentration $\Gamma_{\infty }=3\times 10^{-4}\frac{mg}{cm^{2}}$. The minimum surface tension $\gamma_{min}=4\frac{dyne}{cm}$ occurs at the maximum surfactant concentration $\Gamma_{max}=3.3\times 10^{-4}\frac{mg}{cm^{2}}$. We assumed the relationship between the primary layer surfactant concentration and surface tension is linear from (0, γ_0_) to (Γ_∞_, γ_∞_) and from (Γ_∞_, γ_∞_) to (Γ_*max*_, γ_*min*_) with a slope of *m* and *m*′, respectively. The surface tension equation of state can be expressed as

(20) $$\begin{array}{l} \gamma =\{\begin{array}{cc} m\Gamma_{1}+\gamma_{0}, & {if\;\Gamma }_{1}<\Gamma_{\infty }\\ m^{\prime }\left(\Gamma_{1}-\Gamma_{max}\right)+\gamma_{min} & if\;\Gamma_{1}\geq \Gamma_{\infty } \end{array}, \end{array}$$

where $m=-1.7\times 10^{5}\;dyne\cdot \frac{cm}{mg}$, $m^{{}^{\prime }}=-5.3\times 10^{5}\;dyne\cdot \frac{cm}{mg}$. Figure 5 demonstrates an example hysteresis behavior of alveolar unit as determined from Equations (21) to (25) below.

In Figure 5, $\Gamma_{max}=4.2\times 10^{-4}\;mg/cm^{2}$ is the maximum surfactant concentration, γ_*min*_ = 2 *dyne*/*cm* is the surface tension when Γ_1_ = Γ_*max*_, $\Gamma_{\infty }=3\times 10^{-4}\;mg/cm^{2}$ is the surfactant concentration at equilibrium state, γ_∞_ = 22 *dyne*/*cm* is the surface tension when Γ_1_ = Γ_∞_.

As described by Figure 5, the alveolar unit starts contracting from the maximum volume point (top right corner). During the contraction, the surface tension decreases as the monolayer surfactant concentration increases with simultaneous adsorption/desorption occurring between the surface and bulk phases. When the monolayer surfactant concentration exceeds Γ_∞_, the adsorption from the bulk stops. At this time, the monolayer cannot sustain further compression without collapsing to create a multi-layer with surfactant molecules exuded from the primary to the secondary layer as the alveolar unit continues contracting. Desorption between the primary layer and bulk fluid continues as Γ_1_ exceeds Γ_∞_ and simultaneously the primary layer (Γ_1_) collapses to transport mass to the secondary layer (Γ_2_). At the bottom right corner, the primary layer concentration reaches Γ_*max*_. The speed of collapse increases by an order of magnitude when Γ_1_ > Γ_*max*_. Therefore Γ_1_ ~ Γ_*max*_ as the contraction continues, highly enriching the secondary layer. At the bottom left corner, the alveolar unit starts to expand. The surface tension increases during this expansion due to the decreasing concentration of the primary layer. When the concentration of the primary layer begins to fall below Γ_∞_, respreading starts from the secondary layer to the primary layer, and surfactant molecules are once again adsorbed from the bulk. This hysteresis loop is a key characteristic relating surfactant physicochemical behavior to the stabilizing properties associated with dynamic surface tension in the healthy lung (Notter, 2000).

The governing equations of the surfactant transport are

(21) $$\begin{array}{l} \begin{array}{c} \frac{dM_{1}}{dt}=\left(-j_{c}+j_{r}+j_{a}-j_{d}\right)A\\ \frac{dM_{2}}{dt}=\left(j_{c}-j_{r}\right)A,\\ \frac{dM_{b}}{dt}=\left(-j_{a}+j_{d}\right)A \end{array} \end{array}$$

where *M*_1_ is the surfactant mass in the primary layer, *M*_2_ is the surfactant mass in the secondary layer, *M*_*b*_ is the surfactant mass in the bulk liquid, *A* is the surface area of the air–liquid interface, *j* terms represent the transport between bulk and surface layers. Terms for collapse and respreading are defined as

(22) $$\begin{array}{l} j_{c}=\{\begin{array}{c} C_{collapse}\frac{\Gamma_{1}^{2}}{\Gamma_{1}^{2}+\Gamma_{max}^{2}}max\left(\frac{\Gamma_{1}-\Gamma_{\infty }}{\Gamma_{\infty }},0\right),\;if{\Gamma_{\infty }<\Gamma }_{1}<\Gamma_{max}\\ \exp\left[1000^{*}\left(\frac{\Gamma_{1}}{\Gamma_{max}}-1\right)\right]\cdot C_{collapse}\frac{\Gamma_{1}^{2}}{\Gamma_{1}^{2}+\Gamma_{max}^{2}}max\left(\frac{\Gamma_{1}-\Gamma_{\infty }}{\Gamma_{\infty }},0\right),\;if\;\Gamma_{1}>\Gamma_{max} \end{array},\;and \end{array}$$

(23) $$\begin{array}{l} j_{r}{=C}_{respread}\frac{\Gamma_{1}^{2}}{\Gamma_{1}^{2}+\Gamma_{mls}^{2}}max\left(\frac{\Gamma_{\infty }-\Gamma_{1}}{\Gamma_{\infty }},0\right)\frac{\Gamma_{2}}{\Gamma_{\infty }}, \end{array}$$

where Γ_1_ is the primary layer surfactant concentration, Γ_2_ is the secondary layer concentration, $C_{collapse}=2\times 10^{-4}\;\left(\frac{mg}{s\cdot cm^{2}}\right)$, $C_{respread}=5\times 10^{-2}\;\left(\frac{mg}{s\cdot cm^{2}}\right)$, $\Gamma_{mls}=2.34\;mg/cm^{2}$ is the multilayer respreading concentration (corresponds to γ_*mls*_ = 33 *dyne*/*cm*), $max\left(\frac{\Gamma_{1}-\Gamma_{\infty }}{\Gamma_{\infty }},0\right)$/$max\left(\frac{\Gamma_{\infty }-\Gamma_{1}}{\Gamma_{\infty }},0\right)$ serve as a switch to turn on the collapse/respreading when Γ_1_ is above/below Γ_∞_, and describes the relationship between concentration and speed of transport together with the sigmoidal function $\frac{\Gamma_{1}^{2}}{\Gamma_{1}^{2}+\Gamma_{max}^{2}}$ and $\frac{\Gamma_{2}^{2}}{\Gamma_{2}^{2}+\Gamma_{mls}^{2}}$. The collapse term increases exponentially when Γ_1_ is greater than Γ_*max*_ based on the experimental evidence (Clements, 1957; Horn and Davis, 1975; Schurch, 1995; Krueger and Gaver, 2000). Note that we have modified the collapse and respreading terms from that originally introduced by Krueger and Gaver (2000) in order to stabilize the computations. In the present model we assume that collapse can be initiated (albeit slowly) as soon as the primary layer concentration exceeds Γ_∞_, and this collapse rate increases substantially as Γ_1_ increases to Γ_*max*_. Likewise, respreading initiates when the primary layer concentration falls below Γ_∞_, and the respreading rate increases substantially near Γ_*mls*_.

Terms for adsorption and desorption are defined as

(24) $$\begin{array}{l} j_{a}=\{\begin{array}{c} K_{a}c_{bulk}\left(\Gamma_{\infty }-\Gamma_{1}\right)\left(1-\frac{\Gamma_{2}}{2\Gamma_{\infty }}\right),\;when\;\Gamma_{1}<\Gamma_{\infty }\\ 0,\;otherwise \end{array},\;and \end{array}$$

(25) $$\begin{array}{l} j_{d}=K_{d}\Gamma_{1}\left(1-\frac{\Gamma_{2}}{2\Gamma_{\infty }}\right), \end{array}$$

where *c*_*bulk*_ is the bulk concentration, $K_{a}=1.7\left(\frac{cm^{3}}{mg\cdot s}\right)$, $K_{d}=1.7\times 10^{-2}\left(\frac{1}{s}\right)$, $\left(1-\frac{\Gamma_{2}}{2\Gamma_{\infty }}\right)A$ is the effective surface area.

### Computational Methods

High-performance computing simulations were conducted on Tulane's Cypress supercomputer. The code was constructed in C++ and utilized MPI library. The differential-algebraic equation systems solver used in the code was SUNDIALS IDAS solver with adaptive time stepping (Hindmarsh et al., 2005). The simulation utilizes 8 Intel Xeon E5-2680 v2 CPUs and 128 GB of RAM. Total CPU time for one simulation (180 s of breathing) is ~5 h.

## Results and Discussion

### Overview

In this study, pleural pressure forcing was assumed to be periodic with −5 ≤ *P*_*PL*_ ≤ −8.2 cmH_2_O and a frequency of 12 min^−1^ to simulate tidal breathing (Swan et al., 2012). The inhalation–exhalation time ratio is assumed to be to be 1:2. As an initial condition, the dimensionless liquid film thickness ε, and the total amount of surfactant, *M*_*total*_ = *M*_1_ + *M*_2_ + *M*_*b*_ were prescribed at each airway as well as each acinus when the lung is at its residual volume (RV). In this study, we assumed the minimum alveolar radius equals 100 μ*m*, and the alveolar liquid film thickness equals 0.1 μ*m* at residual lung volume (Bastacky et al., 1985), so ε = 1 × 10^−3^ at RV in the acinus components. For airway components, the initial ε is assumed to be 0.02 (Cassidy et al., 1985). We initially apply a uniform bulk surfactant concentration $c_{aiway}=6\frac{mg}{ml}$ for airway components, and $c_{alveolus}=35\;\frac{mg}{ml}$ for acinar components. During initialization, the equilibrium surface concentrations are determined according to the defined bulk concentration and the physicochemical description above. True surfactant concentration values in the healthy lung are not known, but *c*_*alveolus*_ equals the concentration of the exogenous surfactant Infasurf (ONY, Inc; Amherst, NY).

During the simulation, states of each component are recorded to track the evolution of the system. For visualization, a 3-D figure of the half-lung was generated. This figure reflects the space distribution of each airway and acinus as well as their real-time states. Our simulation was performed for several breathing cycles until stationary states were achieved.

**Figure 6:**
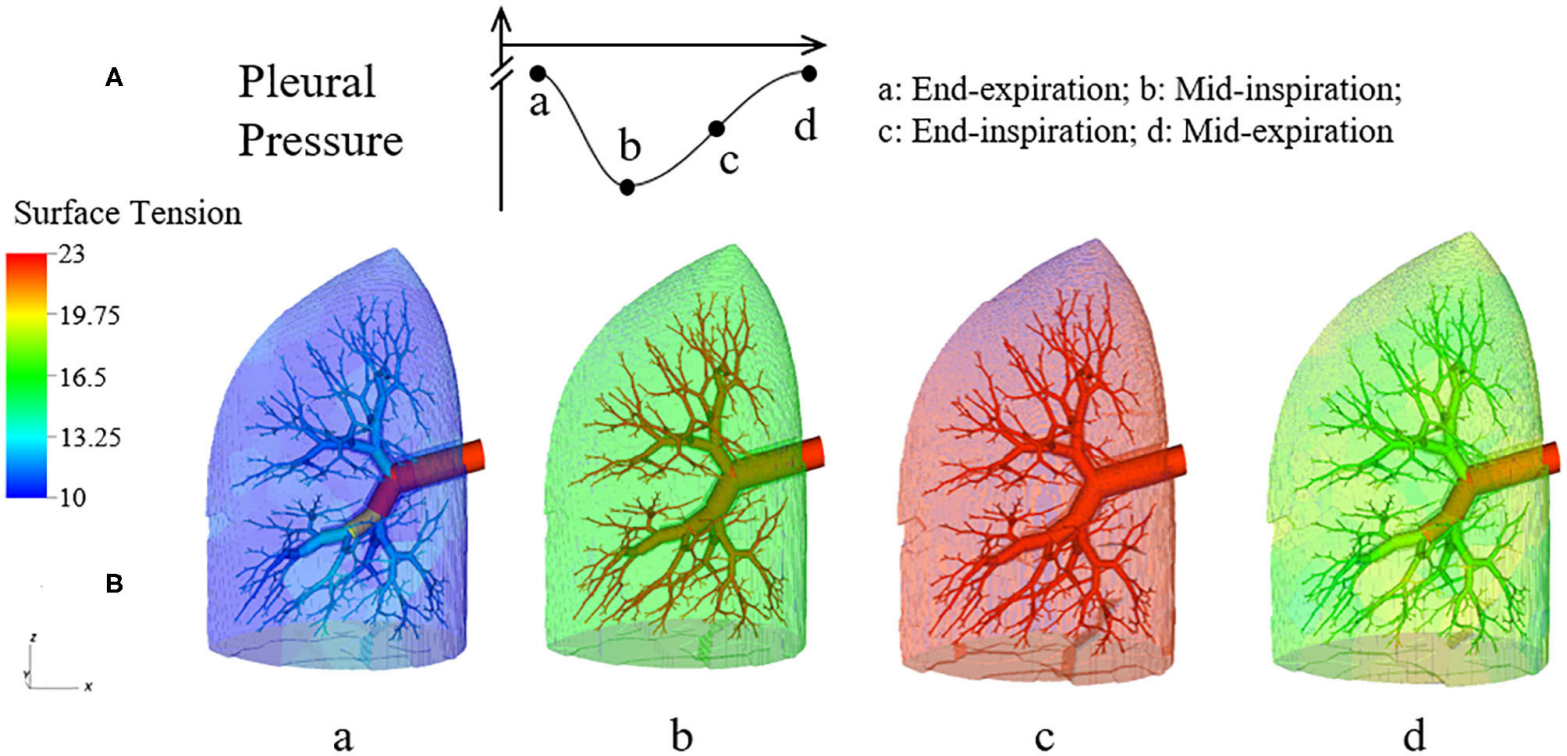
Dynamic surface tension of the lung. **(A)** Specific time points during breathing cycle. **(B)** 3-D figure of the half-lung at four time points. The color scale in the legend denotes the surface tension. Statistical variation is documented in Figure 8.

Figure 6 demonstrates the changes of surface tension in the lung (consisting of airways and acini) at four different time points (a–d) during a breathing cycle. Starting with end-exhalation (a), the surface tension in acini and small airways is as small as 8 dynes/cm, which is far below the equilibrium surface tension (22 dynes/cm) and can be achieved only due to the dynamic transport of surfactant. This low surface tension occurs because the air-liquid interface in each component contracts, increasing the primary layer interfacial surfactant concentration, and decreasing the surface tension (as surfactant also loads the secondary layer). As the lung volume increases during inspiration (b), the surface tension increases, implying a decrease of primary layer surfactant concentration. At the end of inspiration (c), the surface tension attains close to a maximum value in both acini and airways. During expiration (d), the volume of the lung decreases and thus the inner surface area of acini and airways are decreased, reducing the surface tension.

We note that at points a and c, the lung volumes and surface tensions are not respectively the minimum and maximum due to the airway network resistance and the Laplace pressure drop explained in the Pressure–Volume Curve section. Likewise, the lung volumes as well as the surface tensions at points b and d are not identical, even though these represent identical *P*_*PL*_ values. The differentials in lung volume and surface tension are related to the phase-lag that occurs in the system, described below.

### Pressure–Volume Behavior

**Figure 7:**
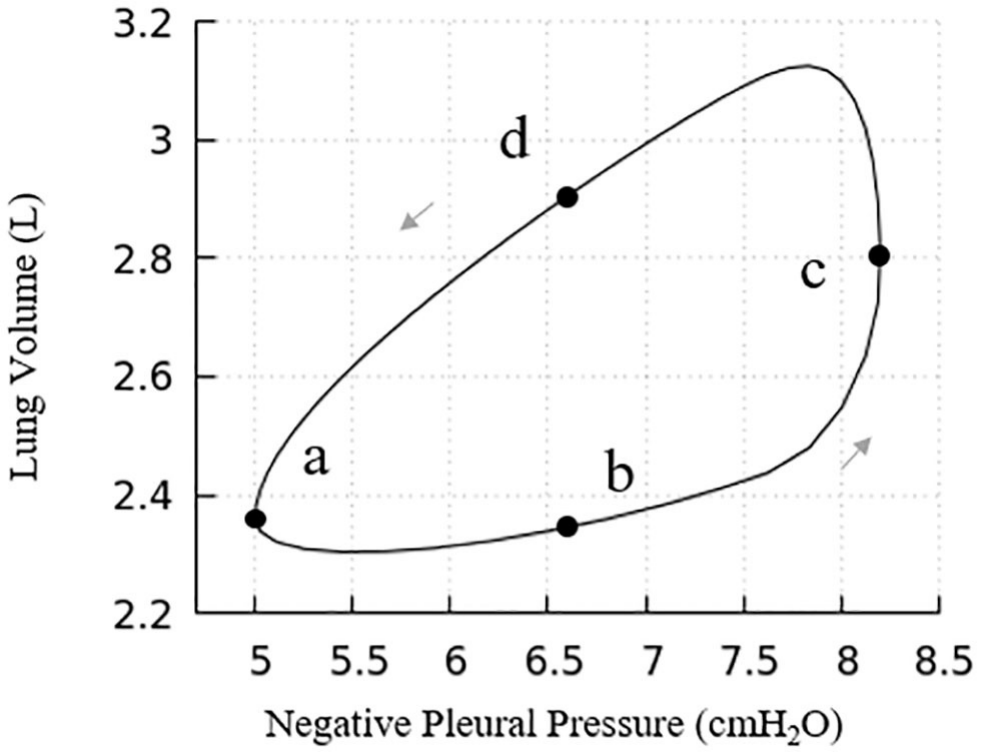
Pressure vs. volume curve of the entire lung. The points (a), (b), (c), and (d) relate to Figure 6. Note, this figure demonstrates a portion of the total PV curve for volumes between end-inspiration and end-expiration for normal tidal volume breathing (~0.82 L).

The pressure-volume behavior described here reflects both lungs, that is twice the size of our computational domain. Our lung model has a full lung residual volume (RV) of 1.0 L. Assuming homogeneous acinar behavior, following Equation (8) the total lung capacity (TLC) is 6.0 L. The dynamic properties of the lung are determined by pressure cycling and Figure 7 presents the pressure-volume (PV) relationship for the entire lung. Under the given pleural pressure range investigated for normal breathing, the tidal volume (TV) equals 0.82 L, with a functional residual capacity (FRC) of ~2.30 L. From these simulations of normal breathing, the maximum flow rate at the trachea is ~0.92 L/s. The average compliance of the lung during tidal breathing is ~0.25 L/cmH_2_O, while the accepted normal value is 0.2 L/cmH_2_O in human lungs (Harris, 2005). TV and compliance of our model is higher than the normal values because the simulated airway resistance is unrealistically low due to the assumptions of laminar, fully developed flow in “Modeling Implementation” as described in “Limitations”. The total airway resistance excluding upper airways ranges from 0.6 to 0.9 cmH_2_O/L/s with lung volume oscillating between 2.30 L (38% of TLC) and 3.12 L (52% of TLC), which is less than the predicted flow resistance at low equivalent lung volumes predicted by Pedley et al. (1970).

Our model presents a typical PV hysteresis behavior, i.e., the compliance of the lung (Δ*volume*/*pressure*) is different between the inspiration and expiration phase. The PV phase lag that is demonstrated in Figure 6 is evident from points a–d in Figure 7.

**Figure 8:**
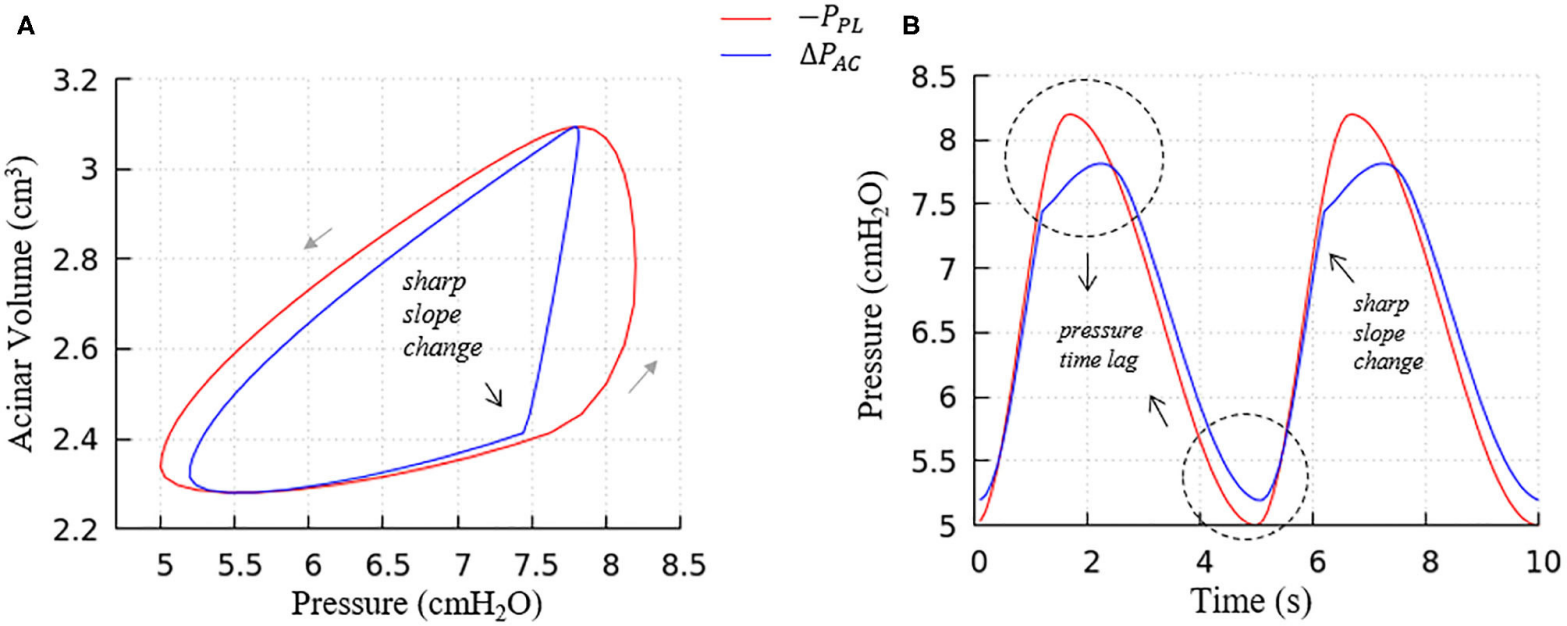
Average acinar global forcing vs. local forcing. **(A)** PV relationship, **(B)** pressure vs. time, global (−*P*_*PL*_, red), local (Δ*P*_*AC*_ = *P*_*AC*_−*P*_*PL*_, blue). Airway resistance leads to the hysteresis area difference between global and local forcing shown in **(A)** and the pressure time lag shown in **(B)**, contributes to the global PV hysteresis behavior.

Figure 8 further elucidates the acinar contributions to PV hysteresis behavior—here we present the behavior in terms of local forcing (Δ*P*_*AC*_ = *P*_*AC*_ − *P*_*PL*_, represented by the blue curve), and global forcing (*P*_*PL*_, represented by the red curve). The global forcing demonstrates a rounded behavior similar to the full lung in Figure 7. However, the behavior of a specific acinus is best understood from the local forcing, Δ*P*_*AC*_.

This analysis demonstrates that the hysteretic behavior is reflected by two factors:

- The flow resistance of airways. As seen in Figure 8A, the hysteresis area of the Δ*P*_*AC*_*V* curve is much smaller than *P*_*PL*_*V*, which results from the network airflow resistance. The time lag between the global and local pressure changes caused by the airway network resistance can be observed in Figure 8B, and causes the lung volume to lag the changes in *P*_*PL*_, as demonstrated by Figure 6.
- The Laplace pressure drop. The hysteresis area of the local PV curve (Δ*P*_*AC*_, represented by the blue curve) results from the behavior of surfactant transport as shown in Figure 5, as described in the next section.

### Surfactant Transport

**Figure 9:**
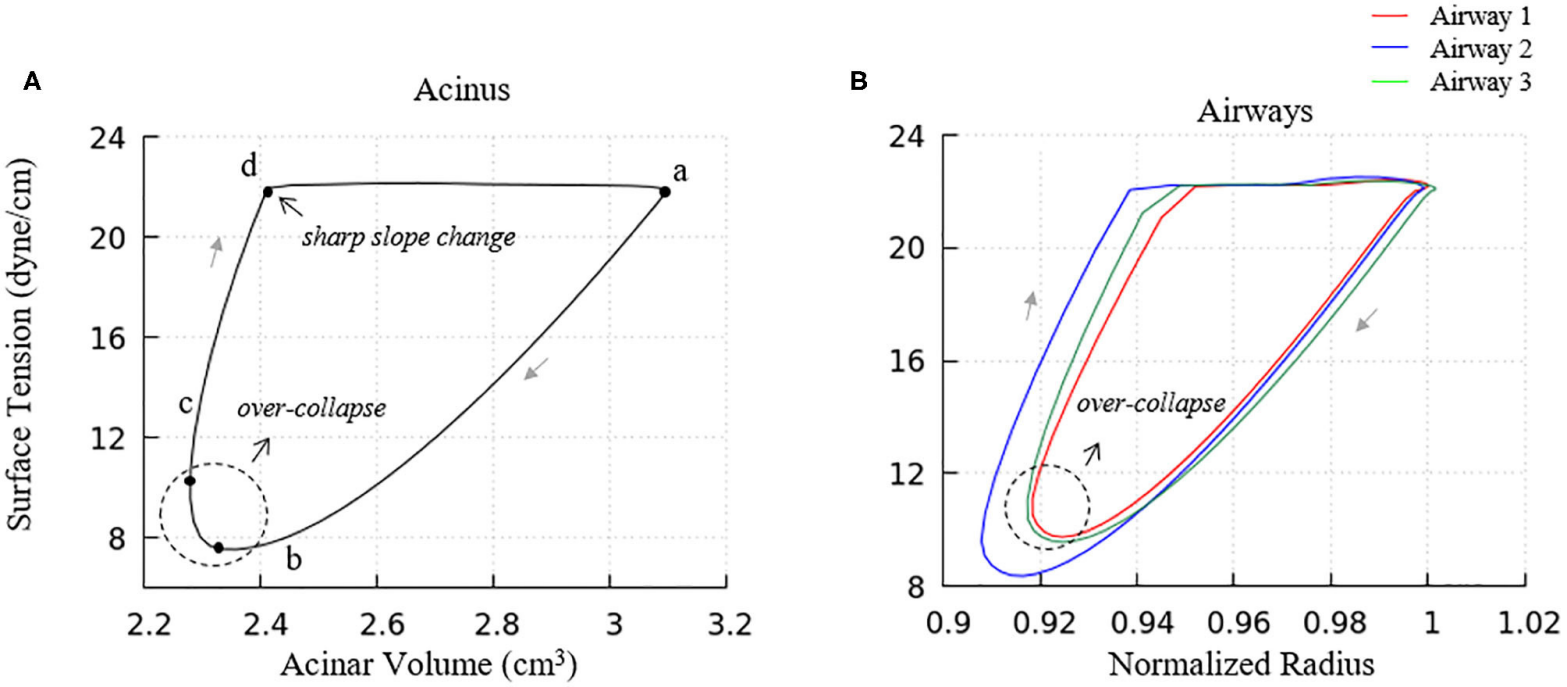
Surface tension behavior of model elements. **(A)** Average surface-tension vs. volume of acinar units. Statistical variation of these element is provided in Figure 10A. **(B)** Representative surface-tension *vs*. normalized surface area of airways demonstrating variations in behavior. The acinar and airway surface tension hysteresis behavior is similar to the isolated model in Figure 5 and contributes to the PV hysteresis behavior of the entire lung. Surface tension variation of airways corresponds to observations in Figure 6.

In order to understand the PV relationships in Figure 8, it is necessary to explore the dynamic surface tension behavior at the alveolar and airway levels (Figure 9). As seen in Figure 9A, the surface tension hysteresis behavior of acinus components is similar to the isolated interface model (Figure 5) except that a minimum surface tension plateau does not exist at the lowest acinar volumes. This deviation occurs because the surfactant concentration of the acinus model does not approach Γ_*max*_ under the breathing conditions imposed by this simulation. In this model, “collapse” refers to the transport of surfactant from the primary to secondary layers. This transport occurs if Γ_1_ > Γ_∞_, and occurs at a greater rate as Γ_1_ increases, to a point where saturation will occur if Γ_1_ ≥ Γ_∞_. In our full-lung model, the rate of interfacial contraction is not large enough to hyper-concentrate the alveolar primary layer to a level that leads to secondary layer saturation. Nevertheless, a significant secondary layer does exist in acinus components.

We note that there is a sharp slope change in the local PV curve in Figure 8A. This abrupt change in compliance is due to the secondary layer respreading, which reduces surface tension and work of breathing. This sharp slope change corresponds to the slope change at point d in Figure 9A.

Airways (Figure 9B) in our model have surface tension hysteresis behaviors similar to acini. Airways exhibit hysteresis areas that varies due to geometrical as well as tethering pressure difference. This corresponds to observations in Figure 6.

We also note that the surface tension increases slightly prior to alveolar and airway expansion (dotted circles), which is likewise observed in the acinar PV relationship (Figure 8A). We attribute this behavior to surfactant over-collapse. In our model, the direction of transport between the primary and secondary layer is only determined by the value of Γ_1_. During most of the phase a–b–c–d in Figure 9A, the primary layer concentration exceeds Γ_∞_, so surfactant transport from the primary to secondary layer occurs. However, as described above, this rate of transport is not large enough to saturate the secondary layer. Nevertheless, the secondary layer is able to respread to the primary layer during the phase d-a in Figure 9A. In phase a– and c–d, the surface concentration is dominated by the surface area change, i.e., the surface concentration increases/decreases as the surface area decreases/increases. However, due to the sinusoidal pleural pressure, the change rate of the surface area in phase c-d decreases dramatically, while the collapse term in Equation (22) does not have any abrupt changes. Here, the surface concentration becomes transport-dominated, i.e., the surface concentration decreases as surfactant being exuded from the primary layer.

**Figure 10:**
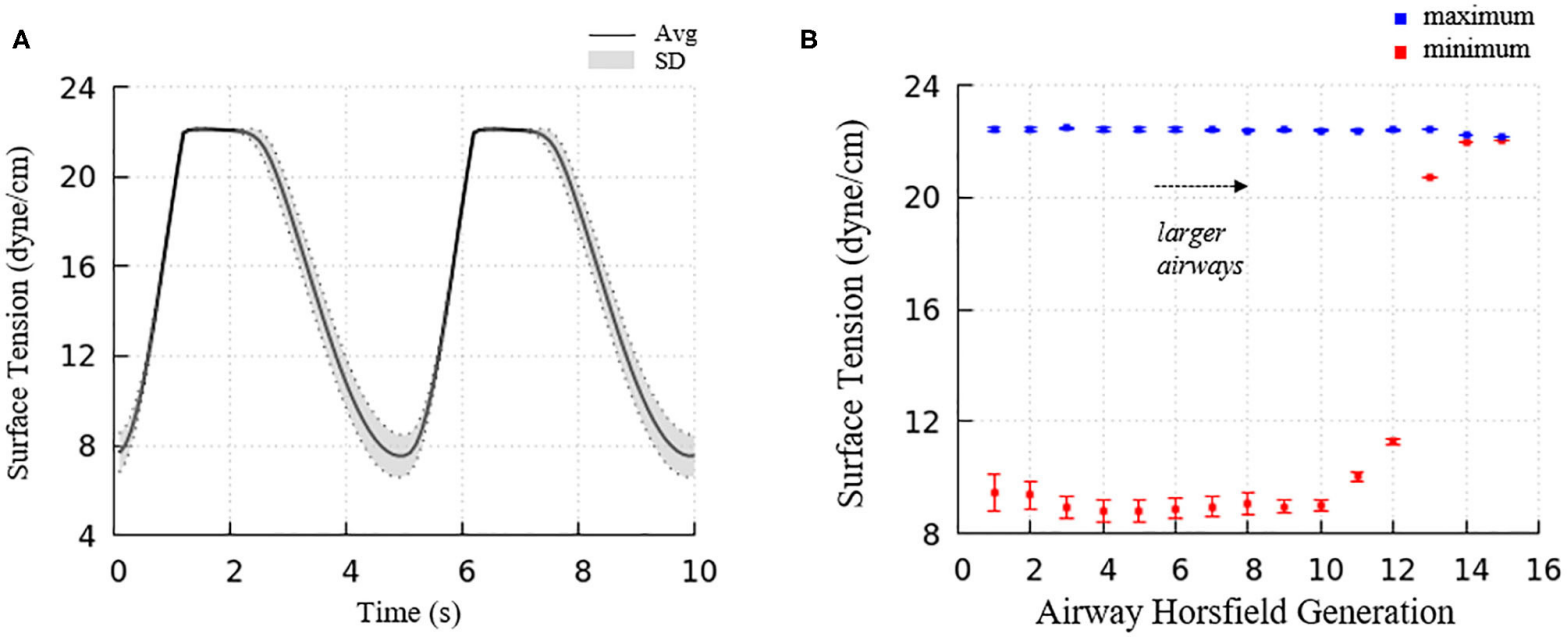
**(A)** Average acinar surface tension with standard deviation. Secondary layer respreading stabilizes surface tension near γ_∞_. Low surface tension demonstrates surfactant collapse behavior. **(B)** The average maximum and minimum surface tension of each airway generation together with standard deviation by Horsfield generation. The minimum surface tension decreases with more compliant (smaller) airways. The secondary layer respreading stabilizes the maximum surface tension near γ_∞_.

Figure 6 demonstrated the surface tension in our model and shows spatial variation. This variation is due to physiologically based heterogeneity that is inherent in our model. For example, there are geometrical differences that exist from generation to generation based upon the space-filling algorithm used to construct the airway tree (Tawhai et al., 2000). Acini are likewise non-uniform because they fill different-sized regions of the lung depending upon the location of the terminal airways. The local surface tension depends upon the lining fluid/surfactant that is distributed within the alveolar components of the acini. While this is assumed to be a uniform concentration, the local strain-field is nonuniform. The physicochemical interactions from variable strain result in transport behavior that leads to non-uniform heterogeneous surface tensions in this dynamic model. Figure 10A demonstrates the average and standard deviations of the acinar surface tension. This figure demonstrates an upper limit of surface tension near γ_∞_ with an insignificant variation. This occurs as the secondary layer respreads, and is indicated by point d in Figure 9A. In contrast, at low surface tension the variation is much greater.

**Figure 11:**
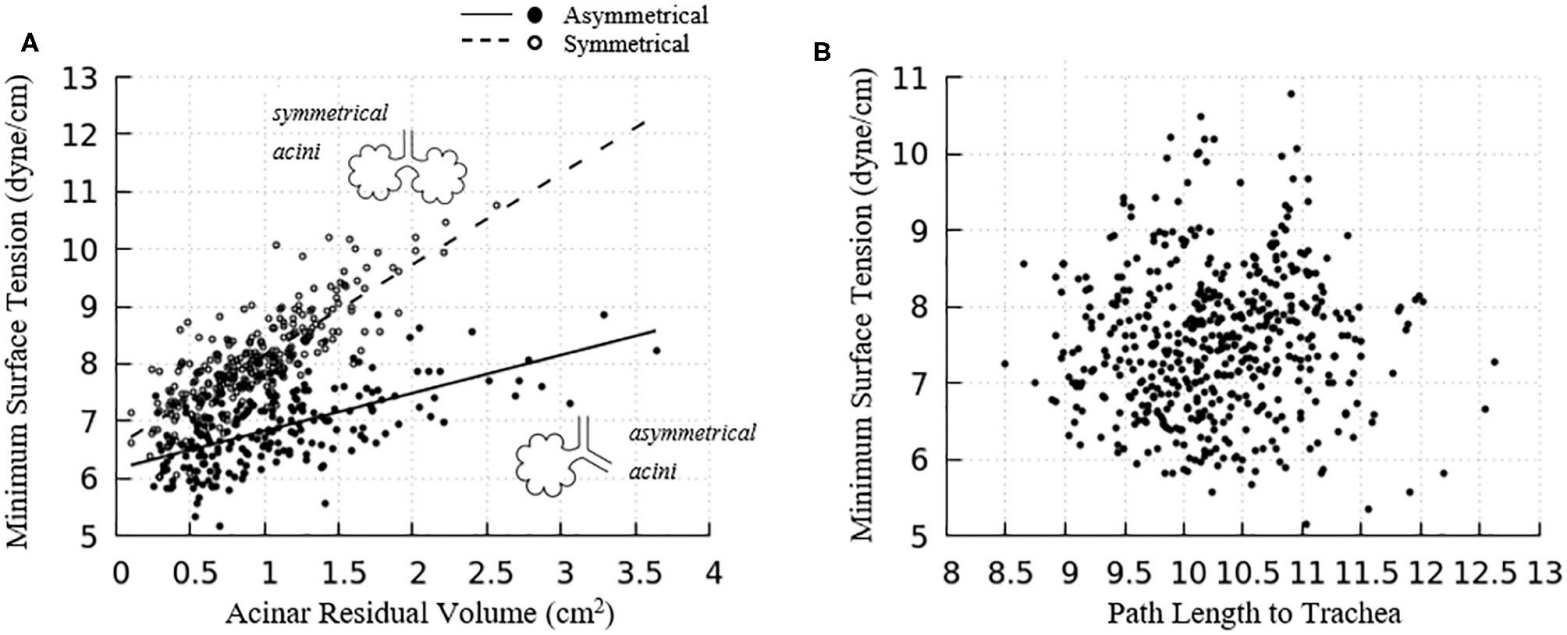
**(A)** Acinar residual volume plotted against the minimum surface tension. Regressions of (1) symmetrical (*a* = 1.58 and *b* = 6.56, reduced chi-square=0.22) and (2) asymmetrical acini (*a* = 0.66 and *b* = 6.16, reduced chi-square = 0.28) show that the surface tension is lower in asymmetrical acini. **(B)** Minimum surface tension plotted against the flow path length to the trachea. No correlation exists.

The acinar minimum surface tension is found to be corelated to its residual volume, as shown in Figure 11A. We subdivide the data into two groups: (1) symmetrical acini that are created at the ends of two equivalent daughter terminal airways, and (2) an asymmetrical acinus that develops from a single terminal airway, with the other daughter airway non-terminal (see figure inset). We investigate the linear regressions using the form γ_*min*_ = *aV*_*RV*_ + *b*. On average, symmetrically produced acini have higher the surface tensions than asymmetric acini. We also explored the influence of central airway-to-acinus path length on the surface tension (Figure 11B) and found no correlation between these values.

For airways, the surface tension heterogeneity also exists. Figure 10B shows the relationship between airway generation (Horsfield) and maximum/minimum airway surface tension. The points represent the average value of all the airways in that generation in one breathing cycle, and the bars represent the standard deviation. Similar to acini, the variation of the maximum surface tension is insignificant due to the surfactant transport mechanism, while a much greater variation of the minimum exists in each generation. The difference among generations can be found to correlate with the tube law defined by Equation (3) and Table 1.

**Figure 12:**
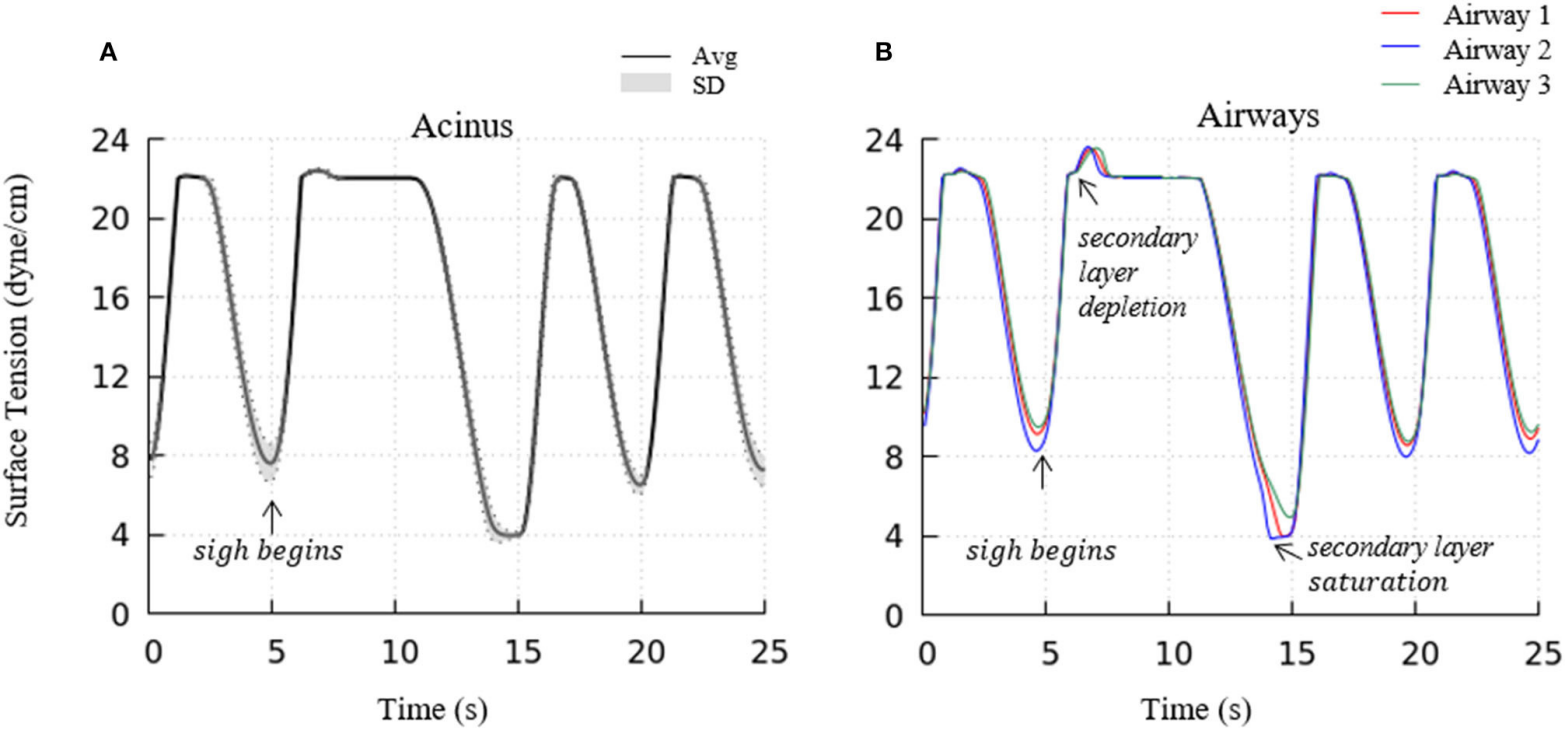
Acinar **(A)** and representative airways, **(B)** surface tension after a deep sigh. The secondary layer respreading stabilizes the surface tension around γ_∞_. In airways, secondary layer depletion can be seen where the surface tension rises rapidly again after being stabilized around γ_∞_ for a short period. Compared to the stationary tidal breathing state, the additional surfactant in the primary layer by respreading from the secondary layer and adsorption from the bulk during sigh leads to a lower surface tension at end-expiration. The representative airways are the same as those shown in Figure 9.

To investigate the impact of a sigh to enhance surfactant surface-layer sorption, we introduced a sigh-like maneuver by temporarily tripling the magnitude of pleural pressure (from 3.2 to 9.6 cmH_2_O), and doubling the time period (from 5 to 10 s per breath). During this cycle, the maximum lung volume increased to 5.96 L. As shown in Figure 12, this high increment of lung volume temporarily depletes the secondary layer and reduces the surfactant concentration of the primary layer, and allows for greater sorption from the bulk to the interface. Upon exhalation, the primary layer is highly concentrated and significantly reduces the surface tension to very low values (<5 dynes/cm). Simultaneously, transfer to the secondary layer occurs, and this leads to a lower surface tension on the following inspiration compared to that observed during the stationary state of tidal breathing.

### Parenchymal Tethering and Strain Deviation

**Figure 13:**
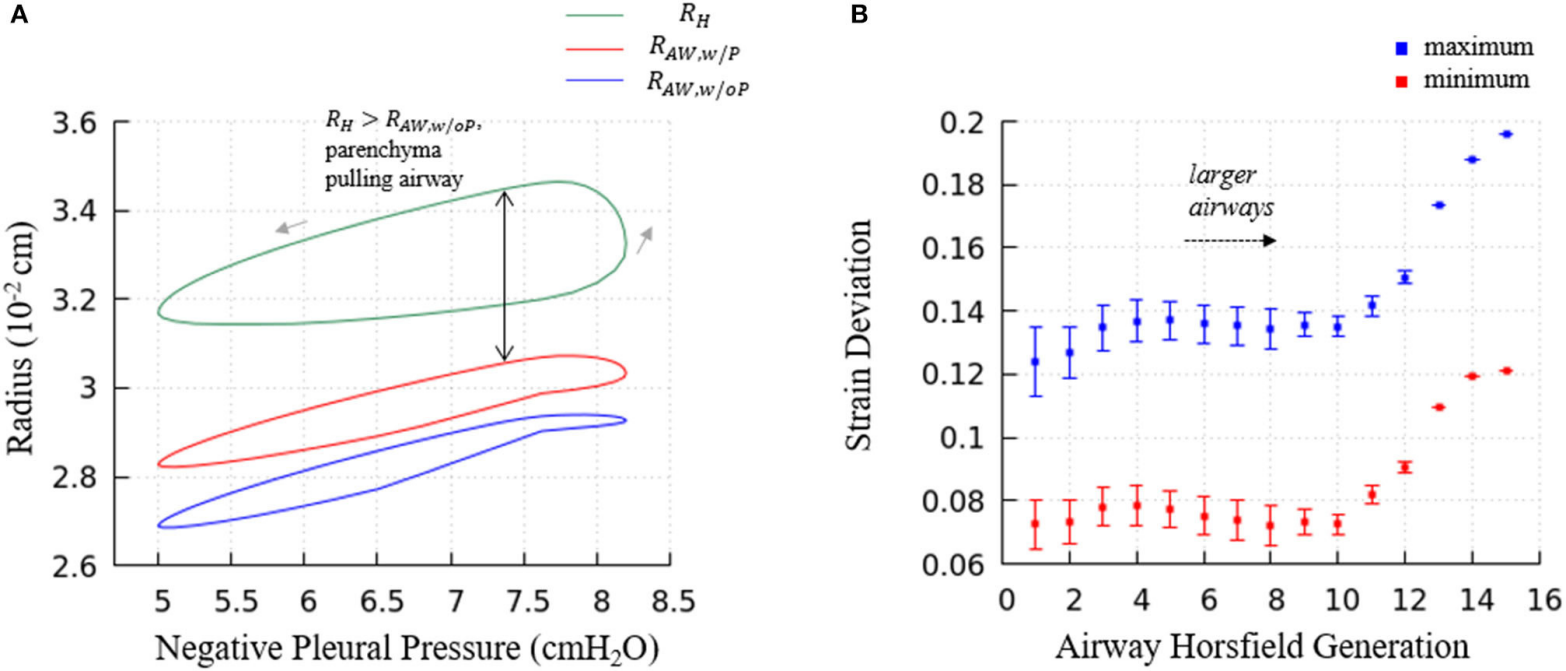
**(A)** Predictions of terminal airway radius with and without parenchymal tethering. Parenchymal hole radius *R*_*H*_ (green), airway radius with parenchymal tethering, $R_{AW,\frac{w}{P}}$ (red), and predicted airway radius without parenchymal tethering, $R_{AW,\frac{w}{o}P}$ (blue). This figure demonstrates the important feature that in this healthy lung model, $R_{H}>R_{AW,\frac{w}{o}P}$. This implies that parenchyma provides an outwardly directed mechanical stress that stabilizes compliant airways. **(B)** The average maximum and minimum strain deviation of each Horsfield airway generation. Bars represent the standard deviation.

**Figure 14:**
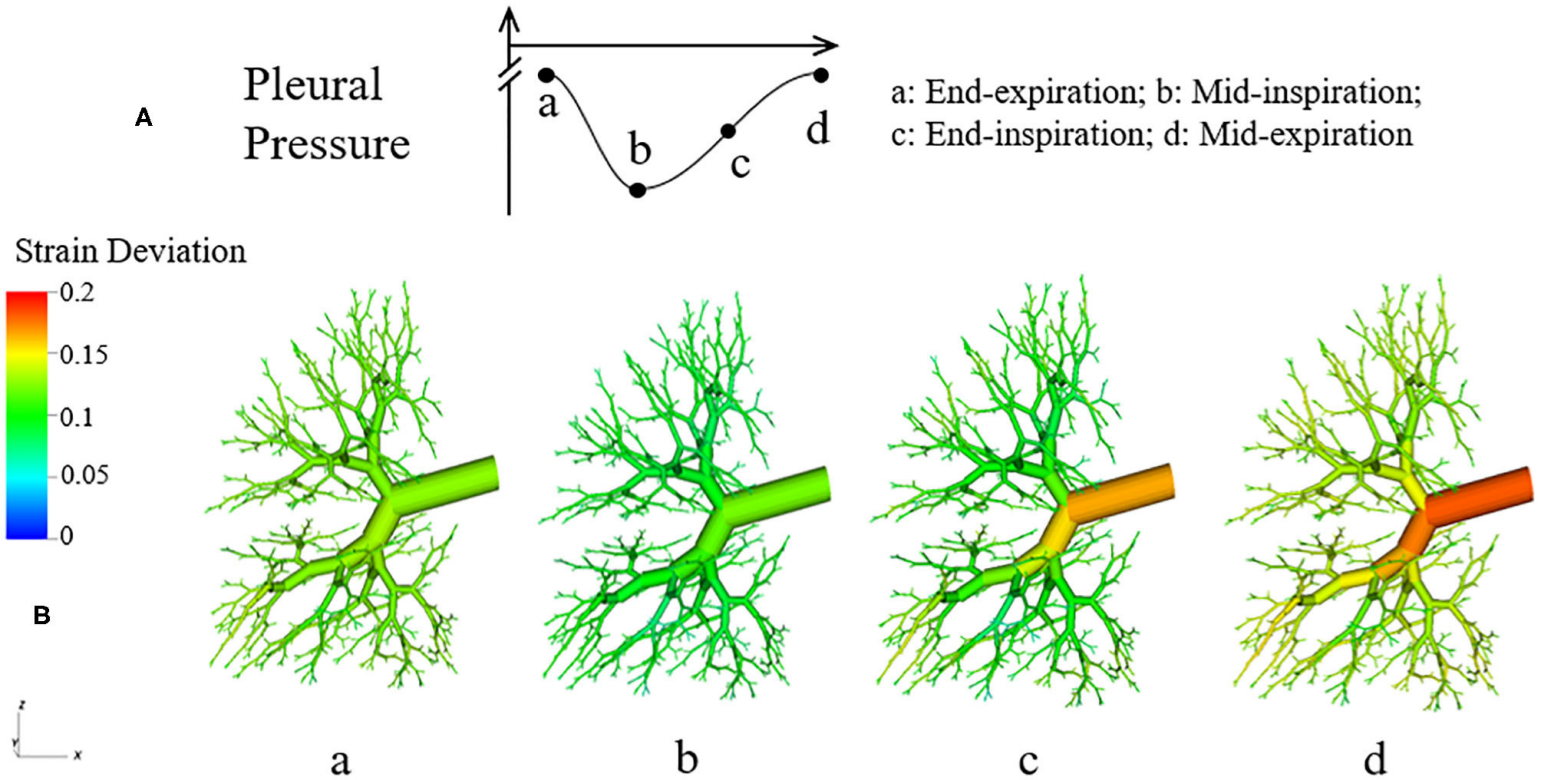
Dynamic strain deviation in the lung model. **(A)** Specific time points during breathing cycle, **(B)** 3-D figure of the airway network at four time points, with color representing the strain deviation from the equilibrium parenchymal state.

As the red curve in Figure 13A shown, airways in our model has a hysteresis pressure-radius behavior similar to the acini. As discussed in the previous sections, this hysteresis behavior is a result from the flow resistance of the airway network and the surfactant behavior (same mechanics as in acini), and more importantly, the pressure-related tethering force. To describe the tethering effect of the parenchyma, we rewrite Equation (12) and define Δ*Strain* as

(26) $$\begin{array}{l} \Delta Strain=\frac{R_{H}-R_{AW}}{R_{H}}, \end{array}$$

where *R*_*H*_ is the hole radius of the parenchyma, *R*_*AW*_ is the airway radius. If Δ*Strain* is negative, it means *R*_*H*_ < *R*_*AW*_, and the tethering force is compressing the airway. Likewise, if Δ*Strain* is positive, *R*_*H*_ > *R*_*AW*_, and the tethering force is pulling the airway open. In Figure 14, the strain deviation at four different time point of the breathing cycle are shown.

It can be observed in Figure 14 that Δ*Strain* is positive almost all the time, which indicates that airways are pulled outward by parenchyma. To elucidate this stabilizing force, we investigate the parenchyma effect $\left[2G_{eff}\left(\frac{\Delta R}{R}\right)\right]$ and airways' own properties $\left[P_{AW}-P_{PL}\left(t\right)\frac{\gamma }{R_{in,AW}\left(t\right)}\right]$ in Equation (7) separately. We simulate the system without tethering, i.e., forcing $2G_{eff}\left(\frac{\Delta R}{R}\right)=0$ in Equation (7).

Figure 13A demonstrates the terminal airway radius with and without tethering ($R_{AW,\frac{w}{P}}$, and $R_{AW,\frac{w}{o}P}\;$, respectively) compared to the size of the hole radius, *R*_*H*_. This figure shows that *R*_*H*_ is greater than the size of the terminal airways. This induces an outward tethering stress that increases the airway radius, so $R_{AW,\frac{w}{P}}>R_{AW,\frac{w}{o}P}$. This stent-like behavior depends upon the set-point for the equilibrium hole-radius (set at *V*_*FRC*_) as described in “Parenchymal Tethering.” This assumption leads to physiologically reasonable positive strain-deviations during normal breathing for nearly all generations of airways, indicating that the parenchymal tethering supports the airway structure in this healthy lung model. We found that reducing the set-point of the equilibrium hole-radius to volumes below *V*_*FRC*_ results in negative-strain deviation during portions of the ventilation cycle for mid-generation and terminal airways (data not shown). This behavior could be destabilizing because it would reduce the caliber of compliant airways and lead to an enhanced proclivity for airway obstruction. This parenchymal support behavior is consistent with earlier simulations conducted by Ryans et al. (2019). The tethering force is important to stabilize the open airway and maintain air flow, though surfactant deficiency can still lead to airway closure or meniscus obstruction in the unhealthy lung (Notter, 2000; West, 2012).

Figure 13B demonstrates the relationship between airway generation (Horsfield) and strain deviation Equation (26). The points represent the average value of all airways in that generation in one breathing cycle, and the bars represent the standard deviation. Small strain deviations exist in the most compliant airways, and this deviation is due to time-dependence in the acinar response related to Equation (17), since the compliance of the airways is approximately equal to the acini. In contrast, for larger airways, significantly positive strain deviation exists due to several factors: (1) a compliance differences between the acinus and airway, (2) the pressure differences associated with large path-lengths between the large airways and surrounding acinus, and (3) the time-dependence in the acinar response.

### Limitations

While this model provides a useful tool for simulating the multi-scale interactions of the lung, there are significant limitations due to the assumed idealized conditions. For example, we neglect gravitational effects and assume a uniform pleural pressure distribution. As such, this model cannot yet simulate ventilation dependency that is linked to orientation. This model also neglects smooth muscle biomechanics that may be neuromodulated. Furthermore, we neglect important pressure losses due to turbulence and entrance-flow effects (Filoche et al., 2015). Our simulations of normal breathing induce a maximum flow rate of ~0.92 L/s in the trachea. The Reynolds number is large (*Re* > 2,000) in airways of generations *n* ≤ 3 and the Womersley parameter indicates unsteady flow in generations *n* ≤ 4. Furthermore, we have assumed fully developed flow, which does not occur near bifurcations. Thus, we can expect that these regions will have greater pressure-drops than those predicted by this model. We also assume that each airway and acinus is initialized with the same dimensionless film thickness ε and a uniform surfactant concentration, and we do not allow for inter-airway or airway-to-acinar transport. Since this is a model of a healthy lung, we do not incorporate interfacial instabilities that can create plugs or regions of atelectasis. Despite these limitations, this multi-scale model a significant step in the development of a multi-scale model of the entire lung, and is capable of reproducing physiologically realistic behavior.

It should be noted that there are a large number of parameters in our system. Some of these are simple physical constants such as viscosity, equilibrium surface tension, adsorption/desorption rates, etc. that can be determined independently. Other parameters include physiological model constants related to the tube law, the set-point for tethering between parenchyma and airways, airway parent-daughter branch angles, etc. Some of these are better known than others, and could be patient specific. These might be considered “tunable” in the sense that they can be subject to revision when more information is available. With present technology, we cannot hope to create patient-specific geometric models of all branching airways and alveoli, lining fluid distributions and regionally dependent surfactant physico-chemical behavior. Despite the inability to define this type of specific information, when fully developed for healthy and pathological states, we will seek to determine the sensitivity of physiological behavior to key measurable parameters. This could provide insight into pathophysiological behavior and system interdependency that is not available from simpler systems. With this understanding, the step toward patient-specific analyses will become clearer.

## Conclusions

We have developed and investigated a computational 3-D lung model that simulates multi-scale pulmonary interactions under healthy conditions. This model includes the airflow between airways and acini, surfactant transport in the liquid lining, and parenchymal tethering between the acini that surrounds airways. As shown above, these simulations demonstrate physiologically realistic macroscale PV relationships and predict microscale strain distributions that deviate from the uniform strain state (Ma et al., 2013). This deviation occurs due to ventilation-induced airflow pressures and non-equilibrium Laplace pressures resulting from surfactant physicochemical interactions (West, 1977, 2012; Krueger and Gaver, 2000; Notter, 2000). In the future, this model will provide a baseline for the study of pathological conditions; for example, airway closure and liquid plug movement that may be important contributors to the etiology of ARDS and ventilator-induced lung injury. Simulations of pathophysiological conditions may foster the development of improved methods of ventilation (Kollisch-Singule et al., 2014; Gaver et al., 2020).

## Data Availability Statement

The datasets generated for this study are available on request to the corresponding author.

## Author Contributions

HM, HF, and DG conceived, designed research, analyzed data, interpreted results of experiments, prepared figures, and drafted manuscript. HM and HF performed experiments. HM, HF, DH, and DG edited, revised manuscript, and approved final version of manuscript. All authors contributed to the article and approved the submitted version.

## Conflict of Interest

The authors declare that the research was conducted in the absence of any commercial or financial relationships that could be construed as a potential conflict of interest.
